# Functional Blockade of Small GTPase RAN Inhibits Glioblastoma Cell Viability

**DOI:** 10.3389/fonc.2018.00662

**Published:** 2019-01-08

**Authors:** Kevin L. Sheng, Kevin J. Pridham, Zhi Sheng, Samy Lamouille, Robin T. Varghese

**Affiliations:** ^1^Fralin Biomedical Research Institute at VTC, Roanoke, VA, United States; ^2^Department of Internal Medicine, Virginia Tech Carilion School of Medicine, Roanoke, VA, United States; ^3^Virginia Tech Center for Drug Discovery, Virginia Tech, Blacksburg, VA, United States; ^4^Wake Forest Baptist Comprehensive Cancer Center, Winston-Salem, NC, United States; ^5^Faculty of Health Science, Virginia Tech, Blacksburg, VA, United States; ^6^Basic Science Education, Virginia Tech Carilion School of Medicine, Roanoke, VA, United States; ^7^Department of Biological Sciences, College of Science, Virginia Tech, Blacksburg, VA, United States; ^8^Department of Biological Affairs and Research, Edward Via College of Osteopathic Medicine, Blacksburg, VA, United States

**Keywords:** RAN, glioblastoma, importazole, cell survival, glioblastoma prognosis, KPNB1, glioblastoma treatment

## Abstract

Glioblastoma, the most common malignant tumor in the brain, lacks effective treatments and is currently incurable. To identify novel drug targets for this deadly cancer, the publicly available results of RNA interference screens from the Project Achilles database were analyzed. Ten candidate genes were identified as survival genes in 15 glioblastoma cell lines. RAN, member RAS oncogene family (RAN) was expressed in glioblastoma at the highest level among all candidates based upon cDNA microarray data. However, Kaplan-Meier survival analysis did not show any correlation between RAN mRNA levels and patient survival. Because RAN is a small GTPase that regulates nuclear transport controlled by karyopherin subunit beta 1 (KPNB1), RAN was further analyzed together with KPNB1. Indeed, GBM patients with high levels of RAN also had more KPNB1 and levels of KPNB1 alone did not relate to patient prognosis. Through a Cox multivariate analysis, GBM patients with high levels of RAN and KPNB1 showed significantly shorter life expectancy when temozolomide and promoter methylation of O^6^-methylguanine DNA methyltransferase were used as covariates. These results indicate that RAN and KPNB1 together are associated with drug resistance and GBM poor prognosis. Furthermore, the functional blockade of RAN and KPNB1 by importazole remarkably suppressed cell viability and activated apoptosis in GBM cells expressing high levels of RAN, while having a limited effect on astrocytes and GBM cells with undetectable RAN. Together, our results demonstrate that RAN activity is important for GBM survival and the functional blockade of RAN/KPNB1 is an appealing therapeutic approach.

## Introduction

Glioblastoma (GBM) is an aggressive tumor generally found in the cerebral hemispheres of the brain. Spanning 16% of the cases of all primary tumors in the brain and ~50% of all malignant brain tumors, GBM is the most common malignant type in the central nervous system (1, 2). The average length of survival for GBM patients is ~15 months, and only about 5.5% of patients will live longer than 5 years after diagnosis and aggressive treatments, such as chemotherapy, radiation therapy, and surgical resection (1, 3–5). However, surgery is not sufficient for a clean and complete resection of the tumor mass due to the infiltration of tumor cells into the normal brain parenchyma. The remaining tumor cells are often refractory to chemo drugs and radiation, thereby contributing to the high incidence of tumor recurrence that is robustly associated with a poor prognosis of GBM (6–9). Therefore, more effective treatments are needed.

To identify novel therapeutic targets for GBM, we and other research groups used RNA interference (RNAi) screening, a technique that allows a simultaneous analysis of genes in a genome for their functions in a particular setting. For example, we performed a genome-wide RNAi screen using a diphtheria toxin negative selection approach (10) and uncovered a molecular pathway that controls the transcription of activating transcription factor 5, a key survival factor for GBM (11). Identification of this molecular survival pathway has led to a phase I clinical trial, in which a combination of radiation and sorafenib, an inhibitor of RAF kinase that suppresses the expression of activating transcription factor 5, was used to treat GBM patients (12). In another study, we carried out a kinome RNAi drop-out screen, through which 20 kinases were identified as survival kinase genes (7). Among these candidates, casein kinase 1 epsilon (13) and phosphatidylinositol-4,5-bisphosphate 3-kinase catalytic subunit beta (14, 15) were further verified as essential survival factors and appealing drug targets for GBM. Studies from other groups have also revealed possible therapeutic targets (e.g., PFKFB4, PLK1, SGK1, NLK, etc.) for GBM using RNAi screens (16–19). Hence, RNAi screening is a proven, useful tool for identifying novel drug targets for GBM.

Recently, the Broad Institute initiated a program termed Project Achilles (20–23). This project aims to complete genome-wide RNAi or CRISPR-Cas9 screens in more than 1,000 different cancer cell lines in order to unveil survival genes in cancer cells and to provide a comprehensive cancer dependency map, allowing for the elimination of tedious and repetitive work of RNAi screens in different laboratories so researchers can further analyze the RNAi screen results to uncover cancer survival genes and develop effective cancer treatments. The principle of these so-called “drop-out” screens is based on the hypothesis that short hairpin RNAs (shRNAs) or guide RNAs (gRNAs) of genes that are essential for cancer cell survival induce cell death; hence, cells with these shRNAs or gRNAs will be depleted over time. By comparing the sequencing reads of shRNAs or gRNAs in cells at the initial and end time point, shRNAs or gRNAs that are lost or under-represented (due to the depletion of cells) will be identified. Results of RNAi screens in more than 500 cancer cell lines, including 15 GBM cell lines, have recently been made available to the public, offering us an opportunity to search for more survival genes in GBM.

In this report, we analyzed RNAi screen results in 15 GBM cell lines and identified 10 candidate genes that are important for the survival of GBM cells. Further comprehensive analyses revealed one gene, RAN (RAN, member RAS oncogene family), as the top candidate because this gene was highly expressed in GBM and its activity was robustly associated with drug resistance and poor prognosis in GBM. RAN is a small GTPase protein that provides energy for nucleocytoplasmic transport and mitotic spindle assembly by hydrolyzing guanosine triphosphate into guanosine diphosphate (24–28). Through this released energy, RAN regulates the activities of the importin protein complexes that mediate nuclear import and export (27–29). Hence, this protein has been implicated in the genesis and disease progression of numerous different types of cancer (30–38). However, the role of RAN in GBM has not yet been extensively explored, despite being shown in two studies as a regulator of apoptosis through blocking Bcl-2-associated X protein and activating survival pathways in GBM cells (39, 40). Directly and selectively targeting RAN is difficult and has not been very successful so far (40, 41). It has been recently shown that importazole, a small molecule inhibitor of RAN and KPNB1, blocks the interactions between RAN and KPNB1 based upon results from fluorescence resonance energy transfer, nuclear localization of fluorescent proteins, and co-immunoprecipitation. The disruption of RAN/KPNB1 complexes represses RAN/KPNB1-mediated nuclear transport (42). We therefore chose importazole to test whether a blockade of RAN activity would inhibit GBM cell viability. While importazole has been tested in different types of cancer, this drug (43–46) has not yet been applied to GBM. We found that blocking the activity of this candidate gene activated cell death and induced a potent inhibition of cell growth in GBM cell lines as well as primary GBM cells, presenting a possibility as an effective drug target for GBM.

## Methods and Materials

### Materials

GBM cell lines, primary GBM cells, and normal human astrocytes were cultured as described previously (7, 14, 47). In brief, GBM cell lines A172, LN-18, SF-268, SF-295, T98MG, U251, and U87MG were maintained in Dulbecco's Modified Eagle Medium (DMEM, Life Technologies) supplemented with 10% EquaFETAL® bovine serum (Atlas Biologicals, Inc.) and 100 μg/ml streptomycin and 100 IU/ml penicillin (Gibco). Primary cells VTC-001, VTC-002, VTC-004, VTC-037, VTC-056, VTC-058, VTC-084, and VTC-103 were cultured in DMEM supplemented with 15% fetal bovine serum (Peak Serum, Inc.) and penicillin/streptomycin. Normal human astrocytes were cultured in MCDB-131 Medium (Sigma) containing 3% fetal bovine serum (Peak Serum, Inc.), 10 X G-5 Supplement (Gibco), and penicillin/streptomycin. Cell lines have been authenticated by the ATCC authentication service utilizing Short Tandem Repeat (STR) profiling. Primary GBM cells were kept at low passages (no more than 10). Antibodies of RAN and GAPDH were purchased from Santa Cruz Biotechnology, Inc. Importazole was purchased from Cayman Chemicals, Inc. Stock solution of importazole was prepared at 50 mM using dimethyl sulfoxide (DMSO). Working solution was further diluted using cell culture media.

### Analysis of RNAi Screen Results From the Project Achilles

RNAi screen results (Achilles_v2.4.6.rnai.gct) were retrieved from the Project Achilles database at the following website: https://portals.broadinstitute.org/achilles. The screen contains more than 50,000 short hairpin RNAs (shRNAs) that target the human genome and the results were presented as fold changes of shRNA loss (log2). The lower the fold change of a particular shRNA, the stronger the depletion of the shRNA in GBM cells. This shRNA depletion is, as hypothesized, due to the loss of cells over time. Results of these shRNAs in 15 GBM cell lines (A172, DBTRG05MG, DKMG, GB1, LN229, LN340, LN382, LN428, LN443, LN464, SF172, SNU1105, U343, U87MG, and YKG1) were first sorted by two or more shRNAs targeting one single gene. More than 4,000 genes were targeted by two or more shRNAs. Next, the fold changes of shRNA loss were averaged. Candidate shRNAs with an average of fold change <-4.0 and a fold change <-3.0 in all 15 GBM cell lines were selected.

### Gene Expression Analyses Using Online Databases

cDNA microarray data were retrieved from BioGPS (http://biogps.org/#goto=welcome), Oncomine (https://www.oncomine.org/resource/login.html), Glioblastoma Bio Discovery Portal (https://gbm-biodp.nci.nih.gov), and The Cancer Genome Atlas (TCGA) database (http://www.cbioportal.org/index.do). Data from BioGPS were reanalyzed. The arbitrary units of mRNAs of candidate genes in GBM cell lines were divided by those in astrocytes, yielding fold changes (GBM/Astrocytes). Regarding data from the Oncomine database, fold changes of candidate gene mRNAs in GBM tissues normalized with those in normal brain tissues from three different studies (Shai Brain, Murat Brain, and Brendel Brain 2) were recorded and summarized in Table 1. *P*-values that determine the statistical significance of fold changes were included as well. mRNA levels of candidate genes in different subtypes of GBM were retrieved from Glioblastoma Bio Discovery Portal. Patient numbers of classical, mesenchymal, and proneural GBM subtypes were 199, 166, and 163, respectively. Levels of candidate gene mRNAs in GBM subtypes were then averaged. RAN levels and MGMT promoter methylation status in GBM patients were retrieved from the TCGA database and were re-analyzed using JMP software.

**Table 1:** Levels of candidate genes in GBM tissues compared to normal brain.

**Gene symbol**	**Shai brain**		**Murat brain**		**Brendel brain 2**	
	**Fold change (GBM/Normal brain)**	***P***	**Fold change (GBM/Normal brain)**	***P***	**Fold change (GBM/Normal brain)**	***P***
NHP2L1	N/A	N/A	1.198	0.006	−2.08	1
PSMB2	2.068	<0.001	1.541	0.007	1.448	0.001
PSMD1	1.226	0.006	−1.328	0.98	−1.001	0.501
RAN	3.375	<0.001	1.265	<0.001	1.512	0.004
RPL23A	1.714	0.006	1.193	<0.001	N/A	N/A
RPS13	1.37	0.003	1.358	0.014	1.278	0.012
RPS15A	1.617	<0.001	1.985	<0.001	1.916	<0.001
RPS7	1.928	<0.001	1.4	<0.001	1.161	0.077
UBB	−1.002	0.505	−1.252	0.992	−1.642	0.994
KPNB1	1.37	<0.001	1.171	<0.001	1.098	0.009
KPNA2	2.063	<0.001	1.67	<0.001	1.608	0.003

*Data were retrieved from the Oncomine database. mRNA fold changes (GBM/normal brain) and P-values that determine the significance of fold changes are shown. Positive numbers indicate more mRNAs in GBM and negative numbers indicate less mRNA in GBM. Candidates showing significantly high levels in GBM in all three studies are highlighted in red*.

### Kaplan-Meier Survival Analysis

Kaplan-Meier survival analyses of GBM patients from the TCGA database have been reported in GlioVis (http://gliovis.bioinfo.cnio.es), Glioblastoma Bio Discovery Portal, and The Human Protein Atlas (http://www.proteinatlas.org). The survival results were retrieved from these databases and presented together with the Log-rank *P*-values.

### Cox Univariate and Multivariate Analysis

Gene expression data and clinical information of GBM patients were retrieved from the TCGA database (http://www.cbioportal.org/index.do). The correlation between mRNA levels and GBM patient survival was determined by Cox univariate or multivariate analysis using the JMP software as previously described (7). Hazard ratios (HR, chance of death) with *P*-values determining HR probabilities larger than Chi-squares were shown. The lower and upper 95% confidence intervals were plotted as well.

### MTS Cell Viability Assay

The MTS cell viability assay was described previously (14, 47, 48). In brief, GBM cell lines, primary GBM cells, and astrocytes were dissociated as single cells and then plated at 500, 1,000, or 4,000 cells per well, respectively, in 100 μl of culture media in a 96-well plate. Next day, cells were treated with importazole at 12.5 μM or at various concentrations (3.125, 6.25, 12.5, 25, or 50 μM, respectively) for 3 or 6 days. A 0.1% DMSO solution was used as the control. At the end point, stock MTS reagent (Promega) was diluted in culture media at 1:10 and added to each well. Two hours later, the absorbance at 490 nm (detecting the color change of MTS in live cells) was measured using a microplate reader (Molecular Devices). Percentages of cell viability were obtained by dividing the MTS readings in importazole-treated cells with those in DMSO-treated cells. *P*-values were determined using the two-way ANOVA.

### Caspase 3/7 Activity Assay

Apoptosis was determined using the caspase 3/7 activity assay as described previously (14, 47, 48). GBM cells and astrocytes were dissociated to single cells and plated at 500 or 4, 000 cells per well in 100 μl of culture media in a 96-well plate. Next day, cells were treated with either a 0.1% DMSO solution or 12.5 μM of importazole. After 3 days, caspase 3/7 activity assay reagent (Promega) was diluted in culture media at 1:1 and added to each well. After 1 h incubation, the luminescence of caspase 3/7 activity reagent was recorded using a microplate reader. Fold changes of caspase 3/7 activity were obtained by dividing luminescence readings in cells treated with importazole with those in cells treated with DMSO. *P*-values were determined using the student *t*-test.

### Immunoblotting

Protein levels were determined using immunoblotting as described in detail previously (14, 47, 49, 50). Briefly, 25–50 μg of total protein was loaded onto an SDS-PAGE gel and then transferred onto a PVDF membrane. Antibodies were diluted as follows: anti-RAN antibody (1:500; Santa Cruz Biotechnology, Inc.), and anti-GAPDH antibody (1:200; Santa Cruz Biotechnology, Inc.).

### Statistical Analyses

Significance of difference in means among different treatment groups was determined using either student *t*-test or two-way ANOVA. The software Prism 7 was used.

## Results

### Analysis of Loss-of Function Screens in GBM Cell Lines

**Figure 1:**
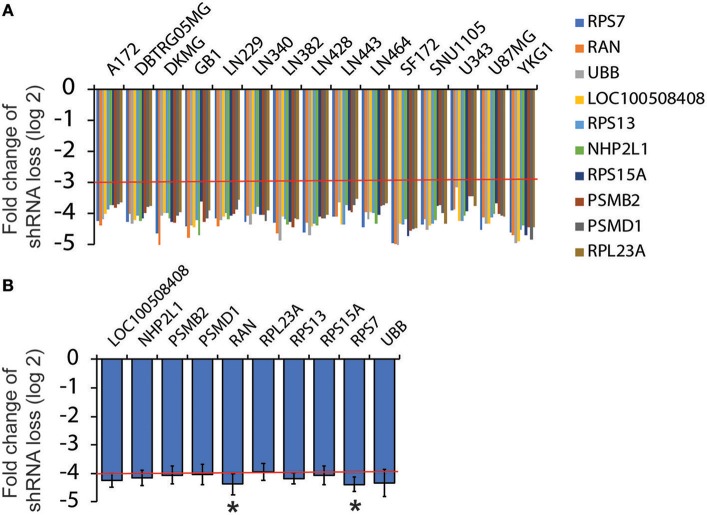
Analysis of RNAi screen results from Project Achilles. RNAi screen results were retrieved from Project Achilles. Candidate genes were selected based on the following criteria: (1) Genes are targeted by two or more different shRNAs; (2) The fold changes of shRNA loss (log2) are lower than −3; (3) The average fold changes of shRNA loss (log2) are lower than −4. Ten candidates were selected. The fold changes of shRNA loss (log2) in each GBM cell lines are shown in **(A)** and the average fold changes of shRNA loss are shown in **(B)**. Red lines indicate cut-off numbers ^*^P < 0.05.

As described earlier, Broad Institute has published drop-out RNAi screens in more than 500 cancer cell lines including 15 GBM cell lines. To identify survival genes from RNAi screens in 15 GBM cell lines, we followed the following criteria: (1) Candidate genes should be targeted by two different shRNAs; (2) The fold change of shRNA loss (log2) should be <−3.0 in every GBM cell line tested (Figure 1A, red line); and (3) The average fold change of shRNA loss (log2) across the 15 cell lines should be below −4.0 (Figure 1B, red line). From more than 50,000 shRNAs, we identified 10 survival genes in GBM.

#### Expression of Survival Genes in GBM

**Figure 2:**
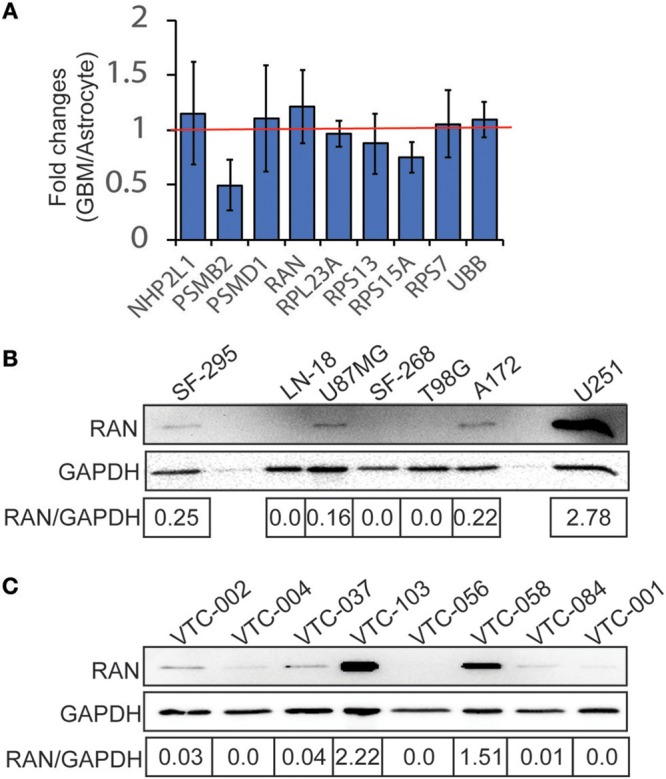
Expression of candidate genes in GBM. **(A)** cDNA microarray data were retrieved from the BioGPS database. The intensities of probes detecting candidate mRNAs in GBM cell lines were divided by those in astrocytes, yielding fold changes (GBM/Astrocyte). Fold changes of NHP2L1, PSMD1, RAN, RPS7, and UBB were above 1 (red line), suggesting that levels of these candidate mRNAs were high in GBM cell lines. Among these candidates, RAN levels in GBM are the highest. Error bars represent standard deviations from six GBM cell lines. **(B)** RAN protein levels in GBM cell lines. RAN and GAPDH proteins were detected in 7 GBM cell lines as indicated using immunoblotting. Band intensities were quantified using Image J. Fold changes (RAN/GAPDH) were obtained by dividing intensities of RAN with those of GAPDH. **(C)** RAN protein levels in primary GBM cells.

Because these genes are important for cell survival, it is likely that they are highly enriched in GBM. To test this possibility, we retrieved cDNA microarray data for GBM cell lines and astrocytes from the online database BioGPS (51–53). By comparing mRNA levels of candidate genes in GBM cell lines and in astrocytes, we found that levels of PSMB2, RPL23A, RPS13, and RPS15A in GBM were lower than those in astrocytes, whereas LOC100508408 was not detected in both GBM and astrocytes. In contrast, mRNA levels of NHP2L1, PSMD1, RAN, RPS7, and UBB in GBM cells were higher than those in astrocytes (Figure 2A, fold change >1.0 as indicated by the red line). Levels of RAN (RAN, member RAS oncogene family) in GBM were the highest among these candidates. We next inquired another online database, Oncomine (54, 55), where tissue microarray results were collected. In three different studies (Shai Brain, Murat Brain, and Brendel Brain 2), fold changes (GBM/normal brain) of RAN, PSMB2, RPS13, and RPS15A were >1 with *P*-values lower than 0.05 (Table 1). In contrast, levels of other candidate genes were not significantly high in GBM. To corroborate the above results, we measured protein levels of RAN in multiple GBM cell lines or primary GBM cells derived from patient specimens (14, 47) using immunoblotting (Figures 2B,C). RAN was detected in SF-295, U87MG, A172, U251, VTC-103, and VTC-058 cells (RAN/GAPDH >0.15; designated as RAN-positive cells), whereas LN-18, SF-268, T98G, VTC-002, VTC-004, VTC-037, VTC-056, VTC-084, and VTC-001 cells did not express RAN or expressed RAN at a very low level (RAN/GAPDH < 0.15; designated as RAN-negative cells). Our results validate the detectability of RAN protein levels in GBM and reveal variations in RAN protein levels amongst GBM cell lines. We have also shown that primary GBM cells proliferate at different rates [see Supplementary Data in (14) for details]. For example, VTC-002 and VTC-103 grew much faster than VTC-056. Intriguingly and consistent with our results shown in Figure 2C, RAN was detectable in VTC-103 and VTC-002, but not in VTC-056. These results suggest that GBM cells expressing RAN have a high-index of proliferation, indicative of a detectable activity of RAN.

### RAN and GBM Prognosis

**Figure 3:**
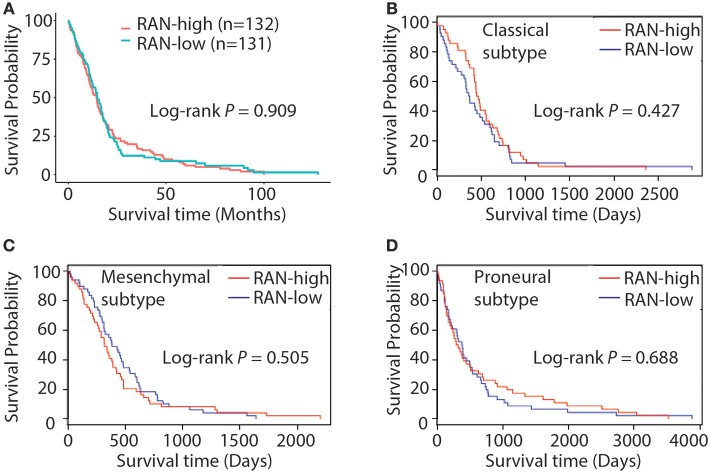
Kaplan-Meier analysis of RAN expression and GBM patient survival. **(A)** Survival curve of GBM patients with different levels of RAN. Results were retrieved from the GlioVis database. Survival curves of classical **(B)**, mesenchymal **(C)**, and proneural **(D)** GBM patients with different levels of RAN were retrieved from the Glioblastoma Bio Discovery Portal. Log-rank *P*-values are shown.

The role of RAN in GBM has not yet been extensively explored. To address this, we tested the hypothesis that RAN, as a survival gene, correlates with GBM patient survival. By querying the TCGA GBM data using The Human Protein Atlas and the Glioblastoma Bio Discovery Portal, we found that RAN levels did not correlate with patient survival (Figure 3A, *P* = 0.909). We further looked into the correlation between RAN and the survival of GBM subtypes and found no statistically significant trend between RAN mRNA levels and the prognosis of classical, mesenchymal, or proneural GBM subtypes (Figures 3B–D, *P* = 0.427, 0.505, or 0.688, respectively).

**Figure 4:**
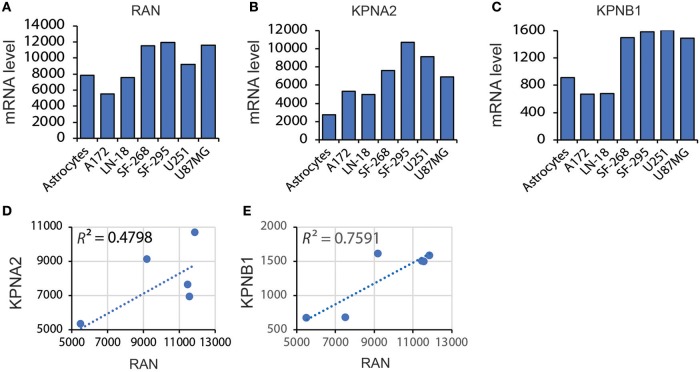
Correlation between levels of RAN and KPNA2 or KPNB1 in GBM. **(A)** Levels of RAN mRNA in astrocytes and GBM cell lines. Data were retrieved from the BioGPS database. The intensities of probes that detect RAN mRNA are shown. **(B)** KPNA2 mRNA levels in astrocytes and GBM cell lines. **(C)** KPNB1 mRNA levels in astrocytes and GBM cell lines. **(D)** Correlation between mRNA levels of RAN and KPNA2 in GBM cell lines. **(E)** Correlation between mRNA levels of RAN and KPNB1. A linear regression model was used. *R* square (*R*^2^) is the coefficient of determination.

These results indicate that mRNA levels of RAN are not associated with GBM prognosis. However, given that nuclear transport is more active in cancer cells due to the high proliferation index (31, 33, 56), the activity of RAN and its functional partners may be more important for GBM survival. RAN regulates nuclear transportation through interacting with importin α, encoded by karyopherin subunit alpha 2 (KPNA2), and importin β1, encoded by karyopherin subunit beta 1 (KPNB1) (57–62). We therefore examined the levels of RAN together with KPNA2 and KPNB1 in GBM cell lines and tissues. Similar to RAN (Figure 4A), mRNA levels of KPNA2 (Figure 4B) and KPNB1 (Figure 4C) were elevated in LN-18, SF-268, SF-295, and U87MG cells. Linear regression analysis revealed that there was a strong trend between levels of RAN and KPNA2 (Figure 4D, *R*^2^ = 0.4798) and levels of RAN and KPNB1 (Figure 4E, *R*^2^ = 0.7591). Congruently, KPNA2 and KPNB1 were also enriched in GBM tissues compared to normal brain tissues (Table 1). In addition, results from the TCGA database showed that levels of RAN, KPNA2, or KPNB1 did not vary among different GBM subtypes (Figure S1).

**Figure 5:**
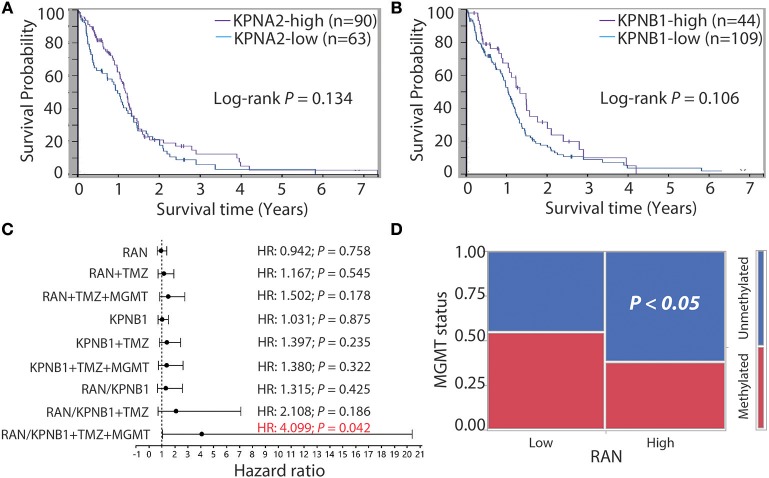
GBM patients with more RAN and KPNB1 exhibit MGMT-dependent TMZ resistance and have shorter life expectancies. Survival curves of GBM patients with different levels of KPNA2 **(A)** or patients with different levels of KPNB1 **(B)** were retrieved from the Human Protein Atlas. Log-rank *P*-values are shown. **(C)** Cox univariate and multivariate analyses of GBM patients with different levels of RAN and/or KPNB1. Data were retrieved from the TCGA database and re-analyzed using JMP software. Hazard ratios (HRs) that determine chances of death are shown. *P*-values indicate the statistical significance of HRs. TMZ treatment (TMZ) and promoter methylation status of MGMT (MGMT) were used as covariates. **(D)** MGMT promoter methylation in GBM patients expressing different levels of RAN. *P* < 0.05 indicates that GBM patients with high levels of RAN often have an unmethylated MGMT promoter.

Next, we determined the relationship between KPNA2 or KPNB1 and GBM prognosis. Based on the TCGA data analyzed using The Human Protein Atlas, we found that levels of KPNA2 (Figure 5A) or KPNB1 (Figure 5B) alone were not significantly correlated with patient survival (*P* = 0.134 and 0.106, respectively), consistent with the results for RAN (Figure 3A). Because levels of RAN and KPNB1 were more closely correlated with each other (Figure 4E), we next interrogated the relationship between levels of RAN and KPNB1 and GBM patient survival. The Cox univariate analysis showed that GBM patients with high levels of RAN or high levels of KPNB1 had a hazard ratio (HR, risk of death) of 0.942 or 1.031, respectively (Figure 5C and Table S1, panel RAN and KPNB1). In contrast, the HR of GBM patients with high levels of both RAN and KPNB1 increased to 1.315 with a *P*-value of 0.425 (Figure 5C and Table S1, panel RAN/KPNB1). To further understand whether this increase suggests a possible link between levels of RAN/KPNB1 and GBM prognosis, we introduced drug resistance into this study as a covariate. Temozolomide (TMZ) is a front-line chemo drug for GBM; however, patients often develop TMZ resistance due primarily to the consequences of promoter unmethylation of O-6-methylguanine DNA methyltransferase (MGMT), an enzyme that repairs TMZ-induced DNA damage (63–65). The poor prognosis of GBM patients is, therefore, closely associated with MGMT promoter methylation. Indeed, GBM patients with high levels of RAN often had an unmethylated MGMT promoter (Figure 5D). We therefore used TMZ treatment (TMZ) and/or MGMT promoter methylation as covariates in a Cox multivariate analysis model. When TMZ was used as a covariate, HRs of GBM patients with high levels of RAN, KPNB1, or RAN/KPNB1 were 1.167, 1.397, or 2.108 with a *P*-value of 0.545, 0.235, or 0.186, respectively (Figure 5C and Table S1, panel RAN+TMZ, KPNB1+TMZ, and RAN/KPNB1+TMZ). By adding MGMT promoter methylation (MGMT) as an additional covariate, HRs of GBM patients with high levels of RAN or high levels of KPNB1 were 1.502 or 1.380 with a *P*-value of 0.178 or 0.322 (Figure 5C and Table S1, panel RAN+TMZ+MGMT and KPNB1+TMZ+MGMT). In contrast, the HR of GBM patients with high levels of RAN and KPNB1 was elevated to 4.099 with a *P*-value of 0.042 (Figure 5C and Table S1, highlighted in red). These results indicate an inverse correlation, associated with MGMT-dependent TMZ resistance, between high levels of RAN and KPNB1 and poor prognosis of GBM patients. Our results together demonstrate that the activity, rather than the expression levels, of RAN is strongly linked to GBM patient survival.

### Functional Blockade of RAN Using Importazole

**Figure 6:**
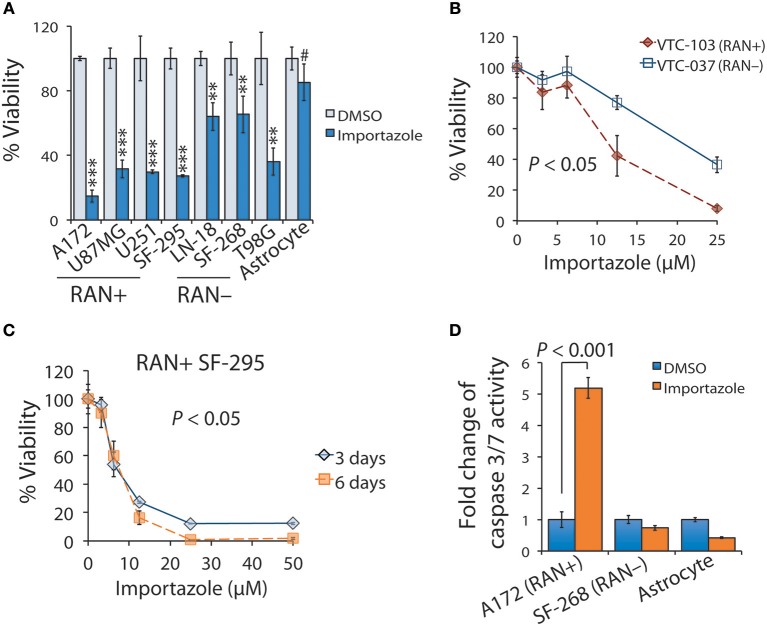
Functional blockade of RAN by importazole induces growth inhibition and activates apoptosis in RAN-expressing GBM cells. **(A)** Viability of GBM cells expressing different levels of RAN and astrocytes when treated with importazole. Cells were incubated with DMSO (light blue bars) or 12.5 μM importazole (dark blue bars) for 3 days. Cell viability was determined using the MTS viability assay. Percentages of viability were obtained by dividing the MTS absorbances of importazole-treated cells with those of DMSO-treated cells. RAN+: RAN-positive; RAN–: RAN-negative. Statistical significance between DMSO and importazole in each cell line was determined using a student *t* test. #*P* > 0.05; ^**^P < 0.01; ^***^P < 0.001. **(B)** Viability of primary GBM cells when treated with importazole. Primary GBM cell lines VTC-103 (RAN+; red line) and VTC-037 (RAN–; blue line) were incubated with importazole at different concentrations ranging from 0 to 25 μM. *P* value that determines the statistical significance between responses of VTC-103 and VTC-037 to importazole at different doses was obtained using a two-way ANOVA analysis. **(C)** Viability of RAN+ SF-295 cells when treated with importazole at different time points. RAN+ SF-295 cells were treated with importazole at different doses ranging from 0 to 50 μM for 3 or 6 days. *P*-value that determines the statistical significance between different time points was obtained using a two-way ANOVA analysis. **(D)** Importazole-induced apoptosis in astrocytes and GBM cells expressing different levels of RAN. Cells were incubated with DMSO or 12.5 μM importazole for 3 days. Apoptosis was assessed using the caspase 3/7 activity assay. Fold changes of caspase 3/7 activity were obtained by dividing luminescence intensities of importazole-treated cells with those of DMSO-treated cells. *P*-value was obtained using the student *t-*test. Standard deviations (error bars) were derived from three independent experiments.

The results shown above suggest that targeting RAN is a potentially appealing approach to impeding GBM disease progression. Our results also indicate that RAN activity in nuclear transport is important for GBM patient survival. We therefore chose importazole to test whether a blockade of RAN activity would inhibit GBM cell viability. We first treated astrocytes and GBM cell lines with 12.5 μM of importazole and monitored cell viability using the MTS cell viability assay. As shown in Figure 6A, importazole decreased the viability of the RAN-positive GBM cell lines A172, U87MG, U251, and SF-295 by >3-fold with a *P* < 0.0001, whereas the RAN-positive GBM cell lines LN-18, SF268, and T98G were much less sensitive to importazole (<3-fold) with a *P* < 0.001. Hence, the statistical analysis shows that the significance of importazole-induced growth inhibition is much stronger in RAN-positive cells (*P* < 0.0001) than in RAN-negative cells (*P* < 0.001). More importantly, importazole only decreased the viability of astrocytes by 15% with no statistical significance (*P* > 0.05). These results suggest that targeting RAN activity is an appealing approach with potentially low side effects. To corroborate these results, we treated RAN-positive or RAN-negative primary GBM cells with importazole at different doses. While both RAN-positive VTC-103 and RAN-negative VTC-037 cells showed a dose-dependent response, VTC-103 cells were more robustly sensitive to importazole than VTC-037 cells (Figure 6B; red line vs. blue line; *P* < 0.05), particularly when cells were treated with importazole at 12.5 or 25 μM. These results were consistent with those obtained from cell lines.

To determine whether importazole response is also time-dependent, we treated RAN-positive SF-295 with importazole at different doses and treatment lengths. We found that the cell viability of a 6-day treatment of importazole was lower than the cell viability of a 3-day treatment, particularly at high doses (Figure 6C). Two-way ANOVA analysis revealed a statistically significant difference between two time points (*P* < 0.05). Hence, the cytotoxic effect of importazole is also time-dependent. Finally, we tested whether the inhibition of cell viability by importazole is due primarily to cell death such as apoptosis. By using the caspase 3/7 activity assay, we found that importazole activated apoptosis, as manifested by the remarkable increase of caspase 3/7 activity in RAN-positive A172 cells, while failing to activate apoptosis in RAN-negative SF-268 cells and astrocytes (Figure 6D). Our results suggest that importazole induces apoptosis in RAN-expressing cells, thereby suppressing cell viability. Taken together, our results demonstrate that a functional blockade of RAN by importazole activates apoptosis in RAN-expressing GBM cells and suppresses GBM cell growth via a time/dose-dependent manner.

## Discussion

**Figure 7:**
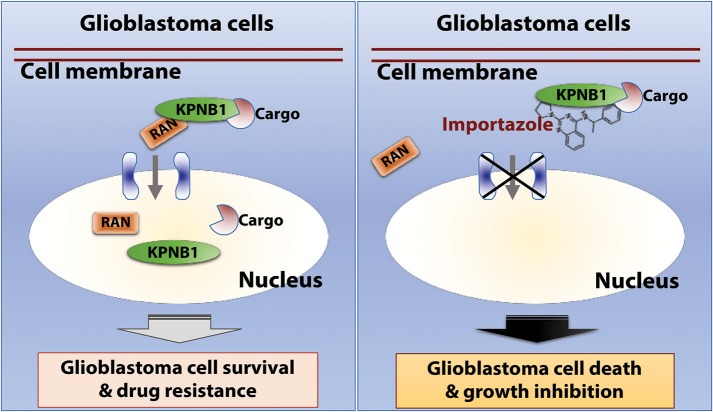
The model of action of RAN in glioblastoma. RAN and its partner KPNB1 regulate nuclear import to promote glioblastoma cell survival and to induce drug resistance in patients **(Left)**. Importazole blocks interactions between RAN and KPNB1, thereby inhibiting nuclear import. The consequences of this blockade are induction of cell death and growth inhibition in glioblastoma **(Right)**.

In this report, we re-analyzed RNAi screen results from Project Achilles and identified RAN as an important survival factor for GBM. Our further investigation of GBM patient data revealed a robust correlation between levels of RAN/KPNB1 and GBM poor prognosis associated with MGMT-dependent TMZ resistance. Moreover, the application of importazole, an inhibitor of RAN/KPNB1 activity, substantially induced cell death and growth inhibition in RAN-expressing GBM cells. Based upon our results together with results from other research groups (31, 33, 56–62), we proposed a model illustrating the mode of action of RAN in glioblastoma (Figure 7). RAN and its partner KPNB1 regulate nuclear import of their cargos to promote glioblastoma cell survival and to induce drug resistance in patients (Figure 7, left panel). Importazole blocks interactions between RAN and KPNB1, thereby inhibiting nuclear import. The consequences of this blockade are the induction of cell death and inhibition of growth in glioblastoma (Figure 7, right panel).

RAN GTPase and proteins involved in nuclear transport have been implicated in cancer progression, drug resistance, and cancer therapeutic development (30, 33, 38, 66). Deng et al., found that RAN was highly expressed in pancreatic cancers with high risk of metastasis (67). Furthermore, depletion of RAN substantially inhibited the migration of metastatic pancreatic cancer cells and the capability of these cells to metastasize to the liver. Congruently, ectopic expression of RAN activates PI3K/AKT signaling and promotes the invasive potential of non-small cell lung cancer cells (36). In a different study, Yuen et al. inactivated RAN in breast cancer cells and significantly increased the sensitivity of these cells to gefitinib (68). The role of RAN and nuclear transport mediated by RAN has not yet been widely explored in glioblastoma. In particular, whether RAN mediates TMZ resistance is not clear. Guvenc et al. examined the expression of RAN and survivin in primary GBM specimens and found that GBM patients with high levels of RAN and survivin were resistant to TMZ (40). They further developed a small chemical compound LLP-3 that disrupted the interaction between RAN and survivin. Incubation of TMZ-resistant GBM cells with LLP-3 diminished TMZ resistance.

These results are consistent with our findings presented above. Our results that demonstrate a strong link between high levels of RAN/KPNB1 and MGMT-dependent TMZ resistance (Figure 5C) are of particular interest. As we described earlier, TMZ is a front-line GBM treatment, but patients often become relapsed despite the reception of TMZ treatment due to the presence of MGMT proteins that repair TMZ-induced DNA damage (63, 69–78). Given that ~45% of GBM patients express MGMT (63), it is therefore critical to overcome MGMT-dependent TMZ resistance. Recent development of MGMT inhibitors has shown modest effect on restoring TMZ sensitivity in MGMT positive GBM patients (79–82). Our findings demonstrate that blocking the activity of RAN/KPNB1 is perhaps an effective approach to enhancing the responsiveness of GBM patients to TMZ, thereby providing a better and more promising therapeutic option for TMZ-resistant GBM patients.

Importazole has also been used in treating other cancers before. Multiple myeloma cell lines RPMI 8226 and NCI-H929 exhibited a strong response to importazole with a 50% inhibitory concentration (IC50) of 4.43 and 4.78 μM, respectively (45). As a comparison, importazole also displayed IC50s at similar range in GBM cell lines and primary tumor cells (Table S2). Given that most cancers, including GBM, demonstrate a hyper-dependency on nuclear transport (31, 44), a selective inhibition of RAN/KPNB1 activity by importazole may represent an innovative and effective treatment for GBM.

While our study unveils the crucial role of RAN in GBM cell survival, important questions remain to be addressed to establish that targeting RAN is an effective treatment option for GBM, particularly those with TMZ resistance. Future studies will reveal whether RAN is a biomarker that predicts MGMT-dependent TMZ resistance in GBM, elucidate how RAN contributes to TMZ resistance, and determine whether importazole or functional blockade of RAN circumvents TMZ resistance and inhibits GBM progression.

## Author Contributions

RTV, SL, and ZS conceived the project and wrote the manuscript. KLS and KJP performed all experiments.

### Conflict of interest Statement

The authors declare that the research was conducted in the absence of any commercial or financial relationships that could be construed as a potential conflict of interest.
