# Supplementary material for: Functional Blockade of Small GTPase RAN Inhibits Glioblastoma Cell Viability
Source: Front Oncol. 2019 Jan 8;8:662. doi: 10.3389/fonc.2018.00662 (PMC6331428; doi:10.3389/fonc.2018.00662)
Supplement: Supplementary file 1 [file Data_Sheet_1.PDF]

Functional blockade of small GTPase RAN inhibits glioblastoma cell viability

Kevin L Sheng[1†], Kevin J Pridham[1†], Zhi Sheng[1, 2, 3§], Samy Lamouille[1, 4, 5§], and Robin T Varghese[6§]

SUPPLEMENTAL MATERIALS

SUPPLEMENTAL TABLES

Table S1. Clinical relevance of RAN and KPNB1 with drug responses and survival of GBM patients.

Analysis	Sample size		Hazard ratio	Lower 95%	Upper 95%	*P*
	High	Low				
**RAN**	80	82	0.942	0.643	1.379	0.758
**RAN+TMZ**	57	53	1.167	0.708	1.931	0.545
**RAN+TMZ+MGMT**	47	44	1.502	0.832	2.769	0.178
**KPNB1**	82	80	1.031	0.706	1.503	0.875
**KPNB1+TMZ**	58	52	1.397	0.806	2.454	0.235
**KPNB1+TMZ+MGMT**	52	39	1.380	0.731	2.641	0.322
**RAN/KPNB1**	29	29	1.315	0.668	2.592	0.425
**RAN/KPNB1+TMZ**	21	26	2.108	0.707	7.106	0.186
**RAN/KPNB1+TMZ+MGMT**	20	12	4.099	1.048	20.383	0.042

Table S2. IC50s of importanzole in RAN-expressing GBM cell lines and primary tumor cells.

RAN-expressing GBM cells	IC50s (μM)
**A172**	4.972
**SF-295**	6.566
**U87MG**	8.755
**U251**	6.651
**VTC-103**	11.38

SUPPLEMENTAL FIGURES

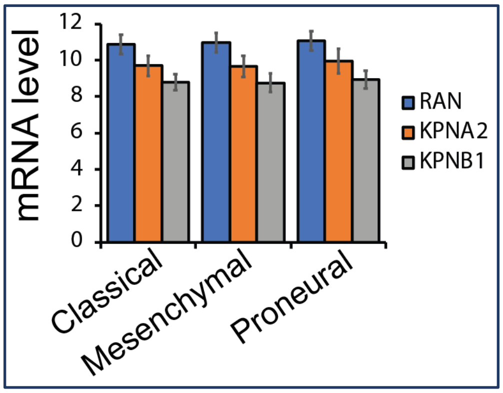

Figure S1. Levels of RAN, KPNA2, and KPNB1 in GBM subtypes. Data were retrieved from the Glioblastoma Bio Discovery Portal. Relative mRNA levels (log 2) are shown.
